# Complete genome sequence and transcriptomics analyses reveal pigment biosynthesis and regulatory mechanisms in an industrial strain, *Monascus purpureus* YY-1

**DOI:** 10.1038/srep08331

**Published:** 2015-02-09

**Authors:** Yue Yang, Bin Liu, Xinjun Du, Ping Li, Bin Liang, Xiaozhen Cheng, Liangcheng Du, Di Huang, Lei Wang, Shuo Wang

**Affiliations:** 1 Key Laboratory of Food Nutrition and Safety, Ministry of Education, Tianjin University of Science and Technology, Tianjin 300457, China; 2 TEDA Institute of Biological Sciences and Biotechnology, Nankai University, Tianjin 300457, China; 3 Department of Chemistry, University of Nebraska-Lincoln, Lincoln, Nebraska 68588, United States

## Abstract

*Monascus* has been used to produce natural colorants and food supplements for more than one thousand years, and approximately more than one billion people eat *Monascus-*fermented products during their daily life. In this study, using next-generation sequencing and optical mapping approaches, a 24.1-Mb complete genome of an industrial strain, *Monascus purpureus* YY-1, was obtained. This genome consists of eight chromosomes and 7,491 genes. Phylogenetic analysis at the genome level provides convincing evidence for the evolutionary position of *M. purpureus*. We provide the first comprehensive prediction of the biosynthetic pathway for *Monascus* pigment. Comparative genomic analyses show that the genome of *M. purpureu*s is 13.6–40% smaller than those of closely related filamentous fungi and has undergone significant gene losses, most of which likely occurred during its specialized adaptation to starch-based foods. Comparative transcriptome analysis reveals that carbon starvation stress, resulting from the use of relatively low-quality carbon sources, contributes to the high yield of pigments by repressing central carbon metabolism and augmenting the acetyl-CoA pool. Our work provides important insights into the evolution of this economically important fungus and lays a foundation for future genetic manipulation and engineering of this strain.

M*onascus* species have been used in food, medicine and industry for over one thousand years^1^. Their most famous fermented product, red fermented rice, has been used extensively as a natural food colorant and food preservative for meat and fish, as a folk medicine to promote cardiovascular health, and as a fermentation starter to brew rice wine and vinegar in China, Japan and other countries in East and Southeast Asia^2^,^3^. In China, colorants produced by *Monascus* have been used to improve the color properties of almost all sausage products (an annual output of more than three million tons)^4^,^5^. It is estimated that more than one billion people eat *Monascus-*fermented products during their daily life.

It is internationally accepted that the genus *Monascus* includes nine species^6^. Based on morphological and physiological analyses, the genus *Monascus* reportedly belongs to the order Eurotiales and the family Elaphomycetaceae^7^ or Monascaceae^8^. However, recent research based on the phylogenetic analysis of four housekeeping genes suggests that *Monascus* should be categorized in the family Aspergillaceae^9^. Due to the lack of a genome sequence, the genetic properties of *Monascus* species, such as their phylogenetic position, have been a subject of debate.

A strong pigment-producing ability is the most outstanding characteristic of *Monascus*. To date, more than 50 different pigments have been identified that are produced by *Monascus*^10^, and many have been shown to possess beneficial functions, such as anti-inflammatory, anti-atherosclerotic, anti-diabetic and anti-cancer activities. Pigments produced by *Monascus* can be divided into three major groups: red pigments (monascorubramine and rubropunctamine), orange pigments (monascorubrin and rubropunctatin), and yellow pigments (monascin and ankaflavin)^11^. Pigment biosynthesis in *Monascus* is believed to follow a polyketide pathway, in which polyketide synthase (PKS) and fatty acid synthase (FAS) have been proposed to play essential roles^12^. Recently, a potential gene cluster (the PKS-FAS gene cluster) involved in the synthesis of pigments was identified in *M. pilosus*, and a homolog of the key PKS gene was experimentally identified by mutagenesis in *M. purpureus*^13^. However, the identities of several other steps and related enzymes involved in pigment biosynthesis are still unclear or remain controversial, and this situation inhibits the construction of industrial strains with better pigment-producing abilities. Additionally, although *Monascus* has been used in food production for more than one thousand years, further investigations of the regulatory mechanisms of pigment biosynthesis and the optimization of fermentation conditions are required. The elucidation of the global regulatory network that controls pigment biosynthesis in *Monascus* will greatly aid such efforts in industry.

*M. purpureus* YY-1 is one of the most extensively used commercial strains in different regions of China for food colorant production. In this study, the whole genome of YY-1 was sequenced using next-generation sequencing (NGS) and optical mapping techniques. The genes involved in pigment biosynthesis were comprehensively investigated for the first time. We performed comparative genomic analysis between *M. purpureus* and other closely related filamentous fungi, which is useful for understanding the evolution and adaptation of *M. purpureus*. We also performed a comparative transcriptomic analysis of YY-1 with high or low pigment yield states, which revealed the regulatory mechanisms underlying pigment biosynthesis in *Monascus*. These advances will guide further studies of *Monascus* species and lay a strong foundation for the highly efficient and modifiable synthesis of useful secondary metabolites.

## Results

### Genome sequence and analysis

**Figure 1:**
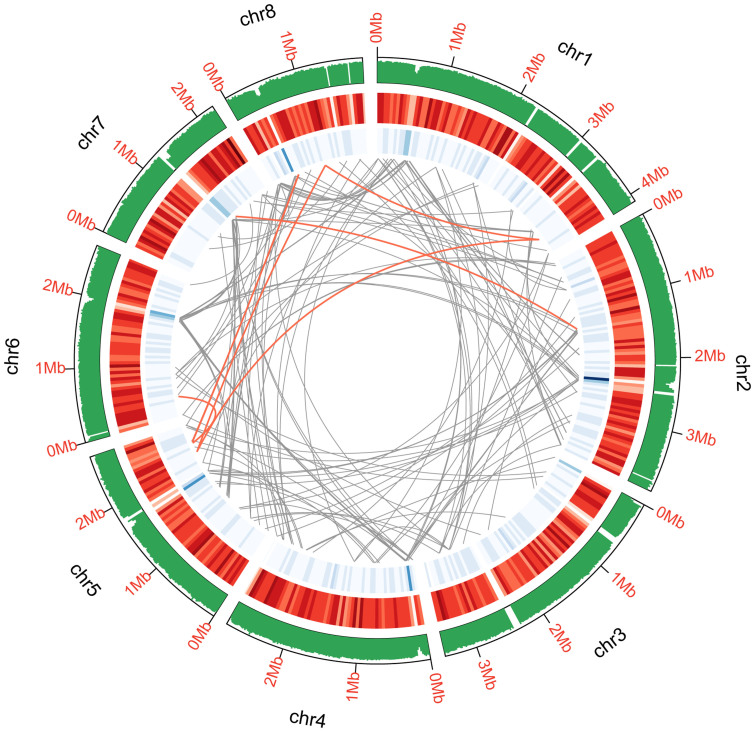
Genomic features of *M. purpureus* YY-1. The tracks from outside to inside indicate GC content calculated as the percentage of G+C in 20-kb non-overlapping windows. Gene density is represented as the number of genes in 50-kb non-overlapping windows (the intensity of the red color correlates with gene density from 0 to 24). Repeat elements were identified by Repeat Modeler with default parameters (blue color intensity correlates with the number of elements from 1 to 56). Genome duplications were calculated by LASTZ with the default option (regions sharing more than 90% sequence similarity over 1 kb are connected by gray lines; those with more than 90% similarity over 2 kb are connected by red lines).

The high-quality genome sequence of *M. purpureus* YY-1 was generated by NGS and optical mapping technologies. This is the first publicly available genome sequence of a *Monascus* species. A genome sequence of 24.1 Mb was obtained by assembling approximately 742 Mb Roche 454 and 3.3 Gb Illumina data (168× coverage). The N50 sizes of the scaffolds and contigs were 1452.0 and 33.292 kb, respectively (Supplementary Table S1). Whole-genome optical mapping technology was used to generate a restriction map of the YY-1 genome, and this analysis ordered and oriented 33 scaffolds (accounting for 98.60% of the genome sequence obtained by NGS) onto eight chromosome-wide superscaffolds (Fig. 1 and Supplementary Fig. S1). Genome annotation identified 7,491 predicted genes, three rRNAs and 124 tRNAs (Table 1).

**Figure 2:**
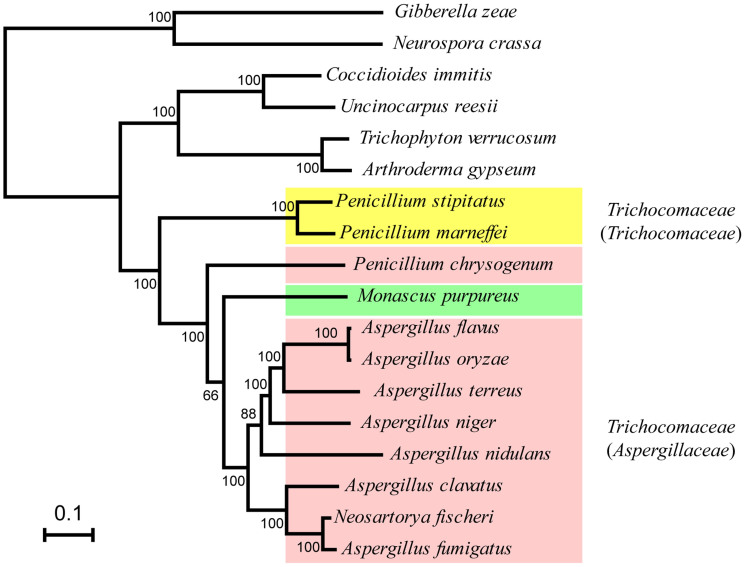
Phylogenetic analysis of *M. purpureus* YY-1. A total of 2,053 single-copy orthologs were selected to construct the phylogenetic tree using RAxML with the maximum-likelihood method. Two *Sordariomycetes* species were selected as an out-group. Species that belong to the traditional family Trichocomaceae and the new family Trichocomaceae are yellow shaded. Species that belong to the traditional family Trichocomaceae and the new family Aspergillaceae are pink shaded. *M. purpureus* is green shaded. Bootstrap values are given at each node.

For filamentous fungi with available whole-genome sequences, 16 species of the class Eurotiomycetes and two species of the class Sordariomycetes (*Neurospora crassa* and *Gibberella zeae*) as an out-group were selected for inclusion in a phylogenetic analysis with *M. purpureus* (Fig. 2). In total 2,053 single-copy orthologs were found. The phylogenetic tree showed that *M. purpureus* exhibits a close relationship with nine species of family Trichocomaceae (Fig. 2). This finding is consistent with previous phylogenetic analyses based on different single DNA loci, which indicated that *M. purpureus* was closely related to *Aspergillus* species. A recent study based on the sequence analysis of four housekeeping genes suggests that Trichocomaceae should be divided into three separate families: Aspergillaceae, Thermoascaceae and Trichocomaceae^9^. In this taxonomic system, our data prove that *M. purpureus* should belong to the family Aspergillaceae, which mainly consists of *Monascus*, *Penicillium* and *Aspergillus*^9^. In summary, our data provide convincing evidence for the evolutionary position of *M. purpureus*.

### Comparative genomic analyses indicate *M. purpureus* has undergone significant gene losses

Comparative genomic analyses revealed that YY-1 has the smallest genome and encodes the smallest number of predicted open reading frames, with a genome size that is 13.6–40% smaller than those of the other 11 sequenced Eurotiales species (Fig. 2 and Supplementary Data S1). However, we found that other genomic features of YY-1, including gene density, average gene length, average number of exons per gene and average exon size, are similar to those of the other sequenced Eurotiales species (Supplementary Data S1), indicating that the small genome size of YY-1 may be attributable to the absence of dispensable genes that were lost during its evolution and adaptation. We investigated paralogous gene families and protein-coding genes among sequenced Eurotiales species (Supplementary Data S2). The data indicated that gene losses in YY-1 represent both missing gene families and decreased numbers of genes within many gene families. It has been suggested that a reduced genome size may convey a selective advantage to microorganisms^14^, which may create substantial efficiencies in energy and nutrient use. It is likely that gene losses have conferred a selective advantage to YY-1 during its evolution, especially with regard to its economic characteristics, such as pigment production.

The KOG classification analysis for YY-1 (Supplementary Fig. S2 and Supplementary Data S3) showed that the number of genes within several categories is comparable to (categories A, B, D, N and Y) or relatively smaller than (categories F, K, O, P and U) those of other sequenced Eurotiales species.

However, it is worth noting that the number of genes in several categories is reduced dramatically in YY-1, primarily genes involved in carbohydrate transport and metabolism (G), amino acid transport and metabolism (E), lipid transport and metabolism (I), and secondary metabolite biosynthesis, transport and catabolism (Q). We found that the missing genes in YY-1 are evenly distributed across the chromosomes, indicating that the gene loss events in the YY-1 genome may have been extensive.

Starch is the major carbon source during the growth of *Monascus* in rice. We found that YY-1 lacks the ability to degrade several oligosaccharides and polysaccharides, such as D-xylan, arabinan and 1,4-β-mannan, because the relevant degradation pathways are incomplete (Supplementary Fig. S3A). Genes encoding β-xylosidase, α-N-arabinofuranosidase and mannan endo-1,4-β-mannosidase are absent in YY-1. Additionally, although some carbohydrate substances can be degraded by YY-1, the efficiency of such degradation is likely decreased, as the number of relevant parallel degradation pathways has been reduced (Supplementary Fig. S3A). For example, there is no cellulose 1,4-β-cellobiosidase II gene, which catalyzes the degradation of cellulose to 1,4-β-D-glucan. Based on the analysis of the carbohydrate metabolic maps for YY-1, *A. oryzae* and *A. fumigatus* (Supplementary Fig. S3A), we found that the number of genes involved in galactose metabolism and the degradation of cellulose, chitin and other glycans is dramatically reduced in YY-1, such as genes for β-galactosidase, β-glucosidase, chitinase and fructose 1,6-bisphosphate aldolase. However, it is interesting that the number of genes with roles in starch metabolism, such as the genes encoding α-amylase (13 genes in total), in YY-1 is almost the same as the number present in the other 11 Eurotiales species (Supplementary Data S4). These findings are consistent with the fact that YY-1 has undergone long-term adaptation to a starch-based food.

YY-1 also has a reduced set of genes involved in amino acid metabolism. We found that although YY-1 is able to synthesize and degrade all types of amino acids, several parallel pathways are incomplete, which may influence the metabolic efficiency. For example, genes encoding 1-aminocyclopropane-1-carboxylate, 4-aminobutyrate aminotransferase, histidine ammonia-lyase, L-3-aminoisobutyrate transaminase, diamine N-acetyltransferase, agmatine deiminase or phenylalanine ammonia-lyase, which are involved in the degradation of L-methionine, L-glutamate, L-histidine, L-valine, arginine and phenylalanine (Supplementary Fig. S3B), respectively, are absent in YY-1, although these amino acids can still be degraded by other parallel pathways. Gene reduction for category E primarily occurred in genes related to the metabolism of tryptophan, tyrosine, phenylalanine, cysteine, serine, methionine, valine, leucine, isoleucine, aspartate, glutamine, glutamate and proline (Supplementary Fig. S3B). Because many amino acids, such as aspartate, asparagine, glutamate, glutamine and serine, are plentiful in rice^15^, the reduction of genes involved in the metabolism of these amino acids should not influence the growth of YY-1.

Fewer lipid metabolism genes are observed in YY-1. The genomes of YY-1, *A. oryzae* and *A. fumigatus* contain 277, 479 and 327 lipid metabolism genes, respectively. In addition to starch and protein, lipids are also a major component of rice. It is reasonable that YY-1 has a limited capacity for lipid biosynthesis. We found that certain parallel pathways for the biosynthesis of fatty acid and other lipids, such as glycerophospholipids and sphingolipids, are incomplete in YY-1. Additionally, the redundancy of genes that play roles in fatty acid biosynthesis, unsaturated fatty acid biosynthesis, fatty acid elongation and steroid biosynthesis was found to be reduced in YY-1. Therefore, the acquisition of acetyl-CoA (a critical substrate for pigment biosynthesis) for fatty acid and lipid synthesis may be reduced in YY-1, which can promote high levels of pigment synthesis.

**Figure 3:**
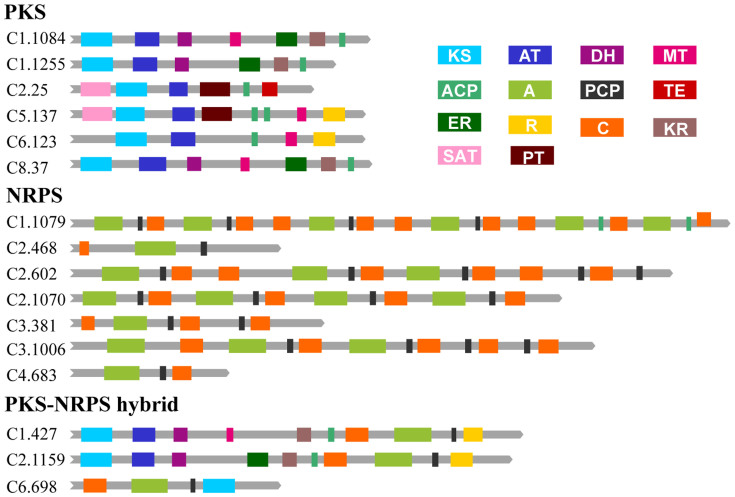
The domains of PKS, NRPS and PKS-NRPS hybrids in *M. purpureus* YY-1. Three types of secondary metabolic genes are grouped together. KS, ketosynthase; AT, acyltransferase; DH, dehydratase; MT, methyltransferase; ER, enoylreductase; KR, keto reductase; ACP, acyl carrier protein; A, adenylation domain; PCP, peptidyl carrier protein; TE, thioesterase; R, reductase; C, condensation domain; SAT, starter unit ACP transacylase; and PT, product template.

Filamentous fungi can secrete a great diversity of secondary metabolites that are synthesized by PKS, NRPS, and the cytochrome P450 superfamily^16^,^17^. The YY-1 genome contains six PKS genes, seven NRPS genes (Fig. 3) and 37 cytochrome P450 superfamily genes—much smaller numbers than those found in the 11 sequenced Eurotiales species, most of which have better capacities for secondary metabolism.

Furthermore, comparative genomic analyses revealed 492 unique genes of YY-1 (Supplementary Data S5). Based on the protein function prediction and transcriptome data described below, we propose that approximately 10% of these unique genes involved in carbohydrate transport and metabolism, oxidation-reduction reaction and NH_3_ unit biosynthesis and release may be related to the pigment yield in YY-1. For instance, α/β-glucosidase (C3.922) and the sugar transmembrane transport protein (C1.1272) may enable YY-1 to use starch efficiently.

### A comprehensive analysis of the *Monascus* pigment biosynthesis pathway

A PKS-FAS gene cluster homologous to that in *M. pilosus* was identified in YY-1. The transcriptome analysis (see below) indicated that under high pigment production conditions, most genes in this PKS-FAS cluster, especially the PKS gene (C5.137), are expressed at high levels (Supplementary Fig. S4 and Supplementary Fig. S5), which further supports the key role of this gene cluster in pigment synthesis. The PKS-FAS gene cluster in YY-1 is somewhat smaller than that in *M. pilosus* (Supplementary Fig. S6), and some genes with unclear function (for instance, 2373-2379 in *M. pilosus*) are absent in YY-1, indicating they are not required for pigment synthesis. The genes between the PKS and FAS genes, which may be the core genes for pigment synthesis, show high conservation. Based on gene function prediction and transcriptomic data, we generated a comprehensive proposal for the biosynthesis pathway of *Monascus* pigment (Supplementary Fig. S7), which provides a strong foundation for the future identification, utilization and modification of relevant enzymes in industry.

Several novel enzymes involved in pigment synthesis were found in our study. Balakrishnan et al. previously suggested that the FAS gene pair (*MpFasA* and *MpFasB*) might be responsible for the biosynthesis of 3-oxoacyl-thioester^13^, which is an intermediate in pigment synthesis; however, no canonical FAS with a similar function has been reported. Other reports suggested that the formation of the condensation product is likely catalyzed by a PKS^18^. We suggest that C5.137 might act as a dual-functioning PKS or that another PKS gene is responsible for the synthesis of 3-oxoacyl-thioester from acyl-thioester. In YY-1, we found another PKS gene cluster (C2.18–C2.32) that may also be related to pigment synthesis. A nonreducing polyketide synthase (NR-PKS) gene (C2.25) in this cluster exhibits a close relationship with four reported pigment biosynthesis genes^19^,^20^,^21^,^22^. Moreover, this NR-PKS shares 54% similarity with a red pigment biosynthesis gene (AAS48892) obtained by complementing an albino mutant in *Nectria haematococca*^23^ (Supplementary Fig. S8). Transcriptome analysis indicated that except for the C5.137 and citrinin PKS C6.123 genes, C2.25 exhibits the highest expression level among PKS genes under high pigment production conditions. We proposed that C2.25 catalyzes the conversion from acyl-thioester to 3-oxoacyl-thioester. Additionally, C2.24 was proposed to be involved in the dehydration reaction of certain steps in pigment biosynthesis (Supplementary Fig. S7). The oxidoreductases (C5.130, C5.135, C1.1074, C6.152 and C7.13) are proposed to reduce the resultant intermediate 11 in the presence of NADPH.

Other pigments, such as yellow pigments, red pigments and water-soluble pigments, are also produced by YY-1. These pigments are derivatives of orange pigments or share related structures with orange pigments. Based on their chemical structures and transcriptomic data, we propose for the first time the candidate genes that may be involved in the synthesis of these pigments (Supplementary Fig. S7). For example, several unique genes in YY-1, such as C6.841, which encodes a CMP/dCMP deaminase zinc-binding protein, and C2.1146 and C4.722, which encode amidohydrolase family protein and n-ethylammeline chlorohydrolase, respectively, may contribute to the release of NH_3_ units. Additionally, the putative aminotransferase gene C1.14 has been proposed to catalyze the amination of orange pigments to generate red and water-soluble pigments.

### A global regulatory network that controls robust pigment production

We found that YY-1 generated the greatest pigment yields when grown in rice medium compared to liquid mediums with other carbon sources, such as malt medium, potato/dextrose medium or sucrose–yeast extract medium (Supplementary Fig. S9). Through experiments using different carbon sources for YY-1, we further confirmed that the pigment yields are significantly increased when rice is used as the sole carbon source in comparison with other carbon sources, such as glucose and sucrose (Supplementary Fig. S10). These findings are consistent with previous reports that different carbon sources can regulate the biosynthesis of secondary metabolism products^10^,^24^. Additionally, we found that during the course of fermentation of YY-1 in rice medium, the biomass increased rapidly in the early logarithmic growth phase (around the fourth day), whereas the pigment value increased rapidly in the late logarithmic growth phase (around the eighth day) (Supplementary Fig. S9); such phenomena have also been reported for other pigment-yielding strains^25^. To investigate the mechanism underlying the high pigment yields during the late logarithmic growth phase in rice medium, transcriptome comparisons were performed for YY-1 grown using different carbon source media (rice medium vs. sucrose–yeast extract medium) and between YY-1 grown for four or eight days in rice medium.

**Figure 4:**
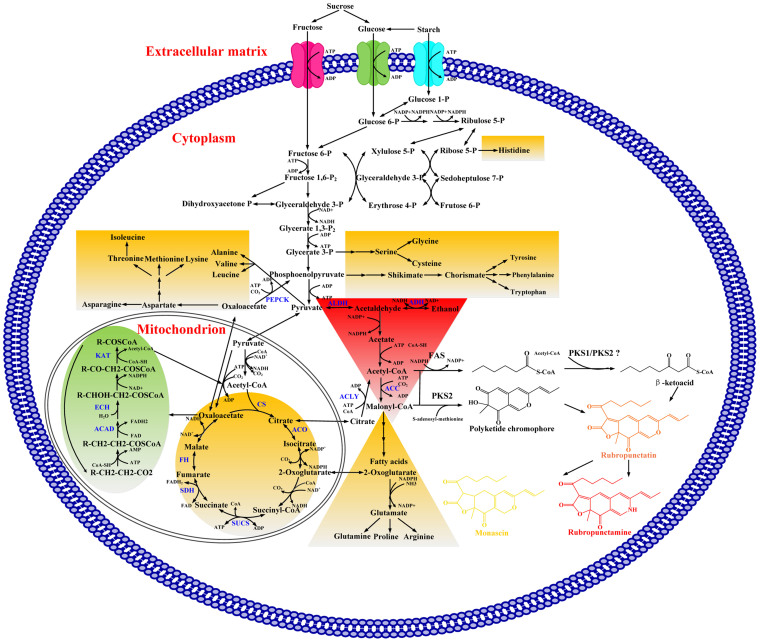
A schematic representation of transcriptional changes in metabolic pathways in *M. purpureus* YY-1 grown with different carbon sources and/or for different growth periods. The transcriptomic changes of several pathways are shown in boxes shaded with different colors. Compared to *M. purpureus* on the eighth day of fermentation in rice medium, red shaded boxes indicate pathways with a notably decreased flux on the fourth day of fermentation in rice medium and on the eighth day of fermentation in sucrose–yeast extract medium; green shaded boxes indicate pathways with an notably decreased flux on the fourth day of fermentation in rice medium but exhibiting only a slight change on the eighth day of fermentation in sucrose–yeast extract medium; yellow shaded boxes indicate pathways with an notably increased flux on the eighth day of fermentation in sucrose–yeast extract medium but exhibiting only a slight change on the fourth day of fermentation in rice medium. The abbreviations in Fig. 4 are as follows: ACLY: ATP-citrate lyase (C5.304 and C5.305), ALDH: aldehyde dehydrogenase (C1.1229, C5.251, C6.127 and C8.272), ADH: alcohol dehydrogenase (C5.130, C2.233 and C3.920), FH: fumarate hydratase (C5.804), ACO: aconitate hydratase (C1.246 and C1.821), CS: citrate synthase (C4.56), SUCS: succinyl-CoA synthetase (C8.356), SDH: succinate dehydrogenase (C3.859, C6.271 and C7.592), PEPCK: phosphoenolpyruvate carboxykinase (C7.128), ACAD: acyl-CoA dehydrogenase (C2.974, C3.771, C4.576, C4.802, C4.895, C5.276, C5.449, C6.66 and C8.265), ECH: enoyl-CoA hydratase (C1.1129, C5.64 and C8.538), KAT: 3-ketoacyl-CoA ketothiolase (C4.541), ACC: acetyl-CoA carboxylase (C7.333).

The transcriptional changes of YY-1 in different media (rice medium and sucrose–yeast extract medium) on the eighth day indicated a complex response of YY-1 to different carbon sources. A total of 1,195 up-regulated and 798 down-regulated genes (the normalized log2R value was ≥2 or ≤2, respectively) were found for YY-1 grown in rice medium. Other than genes without definite predicted functions, these genes are mainly involved in metabolic pathways, such as amino acid metabolism, carbohydrate metabolism and secondary metabolite biosynthesis. The most remarkable transcriptional changes are found in a metabolism network related to acetyl-CoA, which is a crucial metabolite involved in both central carbon and energy metabolism and is also the most important substrate for pigment biosynthesis. Several genes in YY-1 that are involved in two potential pathways for acetyl-CoA biosynthesis are up-regulated in rice medium, such as the genes encoding citrate lyase (C5.304 and C5.305) in the citrate pathway and those encoding aldehyde dehydrogenase (C1.1229, C5.251, C6.127 and C8.272) and alcohol dehydrogenase (C5.130, C2.233 and C3.920) in the acetate pathway (Fig. 4 and Supplementary Fig. S11). Compared to its growth in the rice medium, YY-1 grown in sucrose–yeast extract medium can reach approximately two-fold greater biomass at the late fermentation stage; however, the pigment yield is much lower (Supplementary Fig. S12). Therefore, it is possible that in the sucrose–yeast extract medium, the strain requires more acetyl-CoA to provide sufficient tricarboxylic acid (TCA) cycle intermediates for ATP generation and the synthesis of more amino acids, lipids and fatty acids, which may lead to acetyl-CoA deficiency. In support of these possibilities, the transcriptome data showed that many genes involved in the TCA cycle are up-regulated in sucrose–yeast extract medium, such as fumarate hydratase (C5.804), aconitate hydratase (C1.246 and C1.821), citrate synthase (C4.56), succinyl-CoA synthetase (C8.356) and succinate dehydrogenase (C3.859, C6.271 and C7.592). Additionally, many genes involved in amino acid biosynthesis and fatty acid elongation were also up-regulated in sucrose–yeast extract medium, especially genes responsible for the biosynthesis of aspartate, glutamate, alanine, leucine, chorismate, serine and histidine. Some genes encoding aminoacyl-tRNA synthases (C6.384 and C3.660) were also up-regulated in sucrose–yeast extract medium (Supplementary Fig. S11). In conclusion, compared to the use of sucrose as a carbon source, YY-1 may have shifted the acetyl-CoA metabolic flux to pigment synthesis rather than the cell growth-related pathways when rice was used as a carbon source.

In a comparative transcriptional profile of YY-1 isolated at different fermentation stages (the fourth vs. eighth day) in rice medium, 1,042 differentially expressed genes were identified, of which 527 genes were up-regulated and 515 genes were down-regulated on the eighth day in the rice medium. These results indicated that changes in central carbon metabolism and fatty acid degradation may be the main influences on pigment synthesis.

We found that the acetyl-CoA biosynthetic pathways in the cytoplasm (C5.304, C5.305, C5.130, C5.251) were up-regulated on the eighth day. Additionally, the expression of the phosphoenolpyruvate carboxykinase (PEPCK) gene (C7.128) was up-regulated on the eighth day. As an anaplerotic reaction, the PEPCK branch from the TCA cycle uses a carboxylation reaction that can maintain intermediate pools of phosphoenolpyruvate^26^, which can be converted to pyruvate in the cytoplasm. As high levels of pyruvate can be converted to acetyl-CoA in rice medium, we suggest that this process occurs primarily during the major pigment biosynthesis phase (four to eight days). Therefore, the up-regulation of genes involved in the anaplerotic reaction may indirectly influence carbon flux toward acetyl-CoA and pigment production (Fig. 4 and Supplementary Fig. S13). The up-regulation of the anaplerotic reaction may also slow down the TCA cycle by removing the intermediate metabolites. Because the TCA cycle favors the biosynthesis of biomass components over energy generation, this change in the expression profile may also slow biomass accumulation.

During the course of fermentation, the efficient regulation of fatty acid metabolism allows *Monascus* to acquire various production potentials^12^. Our results indicated that the expression of genes in the fatty acid β-oxidation pathway, such as those encoding acyl-CoA dehydrogenase (C2.974, C3.771, C4.576, C4.802, C4.895, C5.276, C5.449, C6.66 and C8.265), enoyl-CoA hydratase (C1.1129, C5.64 and C8.538) and 3-ketoacyl-CoA ketothiolase (C4.541), increased on the eighth day (Supplementary Fig. S13). The degradation of intracellular fatty acids can enhance the production of acetyl-CoA and malonyl-CoA, which are the precursors of pigment synthesis. In fatty acid biosynthesis, only slight changes in gene expression profiles occurred between the fourth and eighth days, except for that of acetyl-CoA carboxylase (C7.333) (it was up-regulated by 60% on the eighth day), which converts acetyl-CoA to malonyl-CoA. These patterns indicate the enhancement of malonyl-CoA biosynthesis and a slight change in malonyl-CoA consumption for odd-carbon fatty acid biosynthesis, which can provide more malonyl-CoA precursor for pigment biosynthesis.

In summary, the transcriptome analysis and comparisons of the growth curve of YY-1 during fermentation (Supplementary Fig. S12) indicated that carbon flux towards biomass accumulation likely increased in the nutrient-adequate early fermentation stage for YY-1, whereas the strain may favor the biosynthesis of the pigment precursors acetyl-CoA and malonyl-CoA in the moderately nutrient-deficient later fermentation stage, which is mediated by the regulation of acetyl-CoA-related primary metabolism.

### Genes related to the synthesis of other bioactive compounds

Fungal polyketides (PKs) and nonribosomal peptides (NRPs) are two large families of secondary metabolites with remarkable structural diversity and biological activities^27^. Considering the minimal set of domains for PKS^28^ and the crucial set of domains (C) for NRPS^29^, six PKS, seven NRPS and three hybrid PKS-NRPS genes in YY-1 were identified (Fig. 3). The six functional PKS genes are scattered across five different chromosomes, and all are located near telomere regions, implying a high frequency of genetic exchange among these genes (Supplementary Fig. S14 and Supplementary Table S2). Domain prediction analyses indicated that three PKS genes encode NR-PKS and that the other three PKS genes encode highly reducing polyketide synthases (HR-PKS) (Supplementary Fig. S14). The NR-PKS gene C6.123 is likely responsible for citrinin biosynthesis. *Monascus* spp. can be fermented to produce many other beneficial bioactive compounds in addition to pigments^3^, such as ergosterol, γ-aminobutyric acid (γ-GABA) and glucosamine. In YY-1, a gene (C1.1195) was identified that encodes a probable ergosterol biosynthetic protein. Glutamate decarboxylase is the rate-limiting enzyme of a carboxylation reaction that generates γ-GABA^30^,^31^, and three genes (C1.881, C3.567 and C6.424) that encode glutamate decarboxylase were identified. Additionally, three GABA permease genes (C4.511, C5.491 and C6.848) and a predicted gene encoding GABA receptor-associated protein (C6.600) were identified that may contribute to GABA production. A total of 12 genes associated with glucosamine were identified in YY-1, including genes encoding pyrophosphorylase, acetylglucosaminidase, transferase, mutase, isomerase and transporter proteins (Supplementary Data S6). Moreover, a large pool of protease genes (43 in total) is present in YY-1 (Supplementary Data S7), which have great potential for use in the food industry^21^.

## Discussion

In this study, we obtained the whole-genome information of YY-1, which is very useful for further studies on chromosome evolution and genetic variation in *Monascus* and also provides a valuable resource for biological research and industrial applications.

The speciation of *M. purpureus* has been estimated to have occurred approximately 90 million years ago^32^, whereas the first records of the utilization of Hongqu (red yeast rice) in traditional fermentation food and traditional medicine date to approximately one thousand years ago. It is possible or even likely that *Monascus* species have been associated with human life, such as in food production, from a much earlier date. We find that the loss of genes, primarily those that encode components of various metabolic pathways, appears to be a central theme in the evolution of *M. purpureus*, with a clear connection to its adaptation to rice habitats. Similar trends of gene loss are also found in other food-related microorganisms, such as *Streptococcus thermophilus*^33^ and lactic acid bacteria^34^,^35^, which lack many enzymes involved in carbon utilization and the biosynthesis of amino acids due to their adaptation to the milk environment. In contrast, the evolution of *A. oryzae*, a fungus important for the production of traditional fermented foods, has undergone obvious genome expansion^36^ during its adaptation, which might be related to the long-term selection of strains with the ability to produce various flavors in soy sauce.

The response of microorganisms to low carbon source concentrations and quality is similar to the carbon starvation response^37^. Starch, the major carbohydrate component in rice, is regarded as a relatively low-quality sustained-release carbon source because it must be degraded to a monosaccharide or disaccharide before it can be utilized by microorganisms. In submerged shaken cultures, carbon starvation is always observed after the exponential growth phase when the carbon source has been depleted^38^, and the eighth day of fermentation in rice medium represents a common carbon starvation phase. All of these conditions together may lead to carbon starvation stress for YY-1. The carbon starvation stress response is associated with complex physiological, morphological and ultrastructural changes in fungi^38^. Many factors that have been reported to be involved in the carbon starvation stress response were also found to be up- or down-regulated in YY-1 on the eighth day in rice medium, such as the induction of chitinase (ChiB)^39^, N-acetyl-b-D-glucosaminidase (NagA)^40^ and exo-1,3(4)-β-glucanase^41^, which are involved in cell wall metabolism, and dipeptidyl-peptidase^42^, which is involved in nitrogen metabolism, as well as the repression of Catalase A (CatA)^43^ and superoxide dismutase^44^. All of these data confirm the existence of carbon starvation stress in YY-1 on the eighth day of fermentation in rice medium.

The direct and significant influence of carbon starvation on central carbon metabolism has been reported. For instance, genes related to the utilization of glucose from glycolysis to oxidative phosphorylation are reportedly down-regulated under carbon starvation stress in certain fungi, such as *A. nidulans*^38^. Glucose starvation has also been found to repress the synthesis of enzymes involved in central metabolic pathways in *Escherichia coli*^45^. It has been suggested that changes in glycolytic flux might further affect the availability of acetyl-CoA, which can impact secondary metabolite formation^46^. On the eighth day of fermentation in rice medium, when YY-1 likely encountered a high level of carbon starvation stress, the TCA cycle, amino acid biosynthesis and fatty acid elongation, which compete with pigment biosynthesis for precursors, were all down-regulated. In contrast, fatty acid degradation and acetate metabolism, which supply the acetyl-CoA pool, were all up-regulated. In this condition, the strain tends to produce more secondary metabolites, including various pigments. These data suggest that carbon starvation stress can induce pigment biosynthesis by significantly perturbing central carbon metabolism and the acetyl-CoA pool in YY-1. Notably, some pigments have antibiotic activities not only against bacteria but also against yeasts and filamentous fungi^47^. It is likely that significant environmental stress promotes YY-1 to efficiently generate the pigments that can depress other microorganisms.

Few factors related to the regulation of carbon metabolism have been reported in fungi. The global regulatory factor CreA is a famous regulator in fungi that mediates glucose repression in response to environmental cues, mainly carbon source changes^48^. It is interesting that *creA* in YY-1 was also found to be down-regulated in rice medium. However, the comprehensive molecular regulatory mechanisms for pigment synthesis in YY-1 will likely be more complex and include many different regulators; understanding these mechanisms will be the subject of future studies.

## Methods

### Strain and culture conditions

*M. purpureus* YY-1 was obtained from the Fujian Province of China. The pigment-producing abilities of YY-1 in four different liquid media were compared. Media included malt medium (MM) (20 g/liter malt powder, 2 g/liter glucose, 1 g/liter peptone), potato/dextrose medium (PM) (200 g/liter potato, 2 g/liter glucose), rice medium (RM) (20 g/liter rice powder, 20 g/liter glucose, 20 g/liter peptone, 2 g/liter NaNO_3_, 1.5 g/liter KH_2_PO_4_, 1 g/liter MgSO_4_) and sucrose–yeast extract medium (SM) (160 g/liter sucrose, 40 g/liter yeast extract). Dry weight was measured and pigment concentration was determined using a UV-visible spectrophotometer^49^.

To estimate the pigment-producing abilities of YY-1, the carbon source (20 g/liter rice powder and 20 g/liter glucose) of the rice medium (RM) was replaced by different carbon sources: 40 g/liter rice powder, 40 g/liter glucose or 40 g/liter sucrose.

### Genome sequencing and assembly

Two Illumina libraries (0.5 kb and 3 kb) and Roche 454 paired-end (8 kb insert) whole shotgun libraries were constructed for YY-1. Short reads generated from the Illumina paired-end library were assembled using Velvet^50^. Then, contigs were joined into scaffolds with Illumina meta-pair reads and 454 paired-end reads. The completed chromosome-wide sequence pseudomolecules were constructed by anchoring and orienting the final sequence scaffolds onto the whole-genome physical maps of YY-1 that were generated by an optical mapping system.

### Optical mapping and de novo assembly

Optical maps were prepared using Argus (OpGen) according to previously described methods^50^. Briefly, the protoplasts of YY-1 were prepared by enzymatic digestion. High-molecular-weight DNA was prepared by embedding protoplasts in low-melting-temperature agarose plugs followed by treatment with lysing solutions. Genomic DNA was recovered after thoroughly rinsing the plugs in TE followed by melting the plugs at 42°C and subsequent treatment with β-agarase. High-molecular-weight DNA was then immobilized as individual molecules onto Optical Chips, digested with *AflII* restriction enzymes (New England Biolabs, USA), fluorescently stained with a staining kit (OpGen) and positioned onto an automated fluorescent microscope system for image capture and fragment size measurement; these steps resulted in high-resolution single-molecule restriction maps. Collections of single-molecule maps were then assembled to produce whole-genome, ordered restriction maps.

### Gene prediction and annotation

To obtain high-confidence gene models of YY-1, we used an RNA-guided annotation strategy. The prediction of coding genes was accomplished with a modified PASA pipeline using the AUGUSTUS, GlimmerHMM, GeneMark and SNAP algorithms. EVM (Evidence Modeler) was used to merge the preliminary models. All of the predicted gene models were functionally annotated based on their sequence similarity to genes and proteins in the NCBI nucleotide (Nt), non-redundant and UniProt/Swiss-Prot protein databases. The gene models were also annotated based on protein domains using InterProScan. All genes were classified according to Gene Ontology (GO), Kyoto Encyclopedia of Genes and Genomes (KEGG) metabolic pathways and Eukaryotic Orthologous Groups (KOG).

### Orthology and phylogenetic analyses

The peptide sequences were clustered using the Markov clustering program orthoMCL^51^. Peptide sequences were also searched against the nr database using an all-versus-all BLASTp search with a threshold value of E ≤ 1e-5 and were then clustered by MCL with an inflation value of 1.5. Ortholog alignments were produced using MUSCLE (v3.6)^52^ and were then concatenated into a single multiple sequence alignment using an in-house Perl script. A maximum-likelihood tree was reconstructed using RAxML (v5)^53^.

### Secondary metabolite genes and gene cluster prediction

The secondary metabolite biosynthesis gene clusters were predicted with antiSMASH. YY-1 protein sequences were also searched against a local PKS/nonribosomal peptide synthetase (NRPS) database (a subset of the Swiss-Prot database) by BLASTp with a threshold value of E ≤ 1e-5 to identify PKSs and NRPSs. The presence of three hybrid PKS-NRPS genes was confirmed by polymerase chain reaction (PCR) and sequencing of the PCR products (Supplementary Data S8 and Supplementary Fig. S15). The putative PKS/NRPS protein sequences were further searched against the NCBI Conserved Domain Database (V3.09) to confirm that the typical domains were present.

### Transcriptome sequencing and analysis

Total RNA was extracted using the TRIzol extraction method (Invitrogen) according to the manufacturer's protocol. Poly-A mRNA was isolated using oligo-dT-coupled beads from 40 µg total RNA of each sample and then sheared. Isolated RNA samples were used for first-strand cDNA synthesis using random hexamers and Superscript II reverse transcriptase. After end repair and the addition of a 3′-dA overhang, the cDNA was ligated to an Illumina paired-end adapter oligo mix and was size-selected to enrich for ~200 bp fragments by gel purification. After 16 PCR cycles, the libraries were sequenced using Illumina GAIIx. Tophat was used to map mRNA reads to the genome, and Cufflinks were then used to calculate the expected fragments per kb of transcript per million mapped reads (FPKM) as expression values for each transcript.

## Author Contributions

S.W. contributed to the design of the research. Y.Y., B.L., X.J.D., P.L. and D.H. participated in the genome analysis and wrote the manuscript. Y.Y., B.L., X.J.D., P.L., B.L., X.Z.C. and L.C.D. participated in the transcriptome analysis. Y.Y., B.L. and X.Z.C. prepared materials and performed the experiments. Y.Y., B.L., X.J.D., P.L., L.W. and S.W. participated in the coordination and completion of the manuscript. All authors read and approved the final manuscript.

## Additional information

**Accession Codes:** The genome sequence data and RNA-seq data generated in this project are available at http://spxy.tust.edu.cn/duxj/index.html

## Supplementary Material

Supplementary Information Supplementary Information

Supplementary Information Dataset 1

Supplementary Information Dataset 2

Supplementary Information Dataset 3

Supplementary Information Dataset 4

Supplementary Information Dataset 5

Supplementary Information Dataset 6

Supplementary Information Dataset 7

Supplementary Information Dataset 8

## Figures and Tables

**Table 1:** General features of the *M. purpureus* YY-1 genome

Genome assembly length (Mb)	24.1
GC content (%)	49
Number of protein-coding genes	7491
Average gene length (bp)	1744.35
GC content of protein-coding genes (%)	52
Average number of exons per gene	3.19
Average exon size (bp)	491
Average coding sequence size (bp)	1567.49
Average intron size (bp)	82
Average size of intergenic regions (bp)	1701
