# Author Correction: Complete genome sequence and transcriptomics analyses reveal pigment biosynthesis and regulatory mechanisms in an industrial strain, *Monascus purpureus* YY-1

**DOI:** 10.1038/s41598-020-63067-8

**Published:** 2020-04-16

**Authors:** Yue Yang, Bin Liu, Xinjun Du, Ping Li, Bin Liang, Xiaozhen Cheng, Liangcheng Du, Di Huang, Lei Wang, Shuo Wang

**Affiliations:** 1 Key Laboratory of Food Nutrition and Safety, Ministry of Education, Tianjin University of Science and Technology, Tianjin, 300457 China; 2 TEDA Institute of Biological Sciences and Biotechnology, Nankai University, Tianjin, 300457 China; 3 Department of Chemistry, University of Nebraska-Lincoln, Lincoln, Nebraska 68588 United States

Correction to: *Scientific Reports* 10.1038/srep08331, published online 09 February 2015

This Article contains errors.

The sequencing data underlying the study had been previously deposited on the institutional server. The authors have now additionally deposited it in GenBank, under accession code QDGY00000000. The Data Availability section should therefore read:

Data Availability

The genome sequence data and RNA-seq data generated in this project are available at http://spxy.tust.edu.cn/duxj/index.html and in GenBank under accession code QDGY00000000.

