# Supplementary material for: Complete genome sequence and transcriptomics analyses reveal pigment biosynthesis and regulatory mechanisms in an industrial strain, Monascus purpureus YY-1
Source: Sci Rep. 2015 Feb 9;5:8331. doi: 10.1038/srep08331 (PMC4321180; doi:10.1038/srep08331)
Supplement: Supplementary Information [file srep08331-s1.pdf]

Complete genome sequence and transcriptomics analyses reveal pigment biosynthesis and regulatory mechanisms in an industrial strain, *Monascus purpureus* YY-1

Yue Yang[1†], Bin Liu[2†], Xinjun Du[1†], Ping Li[1], Bin Liang[1], Xiaozhen Cheng[1], Liangcheng Du[3], Di Huang[2], Lei Wang[2], ShuoWang[1*]

[1] Key Laboratory of Food Nutrition and Safety, Tianjin University of Science and Technology, Tianjin 300457, China

[2] TEDA Institute of Biological Sciences and Biotechnology, Nankai University, Tianjin 300457, China

[3] Department of Chemistry, University of Nebraska-Lincoln, Lincoln, Nebraska 68588, United States

[†] These authors contributed equally to this work and should be treated as co-first author.

*Corresponding Author: Dr. ShuoWang: s.wang@tust.edu.cn

Supplementary Tables

Supplementary Table S1. Statistical characteristics of the *M. purpureus* YY-1 genome assembly

Large scaffolds (>1000 bps):		Large contigs (>1000 bps):	
Largest scaffold	C1	Largest contig	
Largest length	4,109,886	Largest length	166,030
No. of large scaffolds	33	No. of large contigs	1102
Bases in large scaffolds	24,147,356	Bases in large contigs	22,216,052
N50 scaffold	4	N50 contig	208
N50 length	2,821,034	N50 length	32,919
N90 scaf	7	N90 contig	669
N90 length	2,248,774	N90 length	9814
GC content	49.14%	GC content	49.14%

Supplementary Table S2. Polyketide synthases of *M. purpureus* YY-1: gene location, orthologs and putative functions

PKS	chromosome location	Size (bp)	Closest matches	Similarity %	Type & putative function/product in other fungi
PKS 1	Evm.model.C1.1084	8388	*Aspergillus clavatus* XP_001270320.1	78	Polyketide synthase
			Colletotrichum gloeosporioides XM_007272514.1	71	Polyketide synthase
			Magnaporthe oryzae XP_003717383.1	67	Fatty acid synthase S-acetyl-transferase
PKS 2	Evm.model.C1.1255	9034	*Penicillium chrysogenum* XM_002567484.1	70	Hypothetical protein
			Penicillium chrysogenum AM920436.1	68	
			Penicillium marneffei HM070051.1	68	Polyketide synthase (PKS7)
PKS 3	Evm.model.C2.25	6535	*Monascus purpureus* AJ414729.1	99	Polyketide synthase (pks1)
			Monascus purpureus AY954028.1	99	Polyketide synthase (PKS1)
			Vulpicida pinastri AB267849.1	74	Polyketide synthase
PKS 4	Evm.model.C5.137	8144	*Monascus purpureus* JX866749.1	99	Transcription factor and non-reducing polyketide synthase

			Monascus pilosus KC148521.1	97	Pigment biosynthetic gene cluster
			Penicillium marneffei HM070047.1	67	Polyketide synthase (PKS3)
			Penicillium marneffei XM_002149733.1	67	Polyketide synthase
PKS 5	Evm.model.C6.123	9140	*Monascus aurantiacus* EU309474.1	100	Citrinin biosynthesis gene cluster
			Monascus purpureus AB243687.1	100	Citrinin biosynthesis
			Monascus purpureus AB167465.1	100	Citrinin polyketide synthase (pksCT)
PKS 6	Evm.model.C8.37	8519	*Monascus purpureus* AY312161.1	99	Like polyketide synthase
			Monascus purpureus AY312162.1	99	Like polyketide synthase
			Monascus purpureus AY312164.1	98	Like polyketide synthase

Supplementary Figures

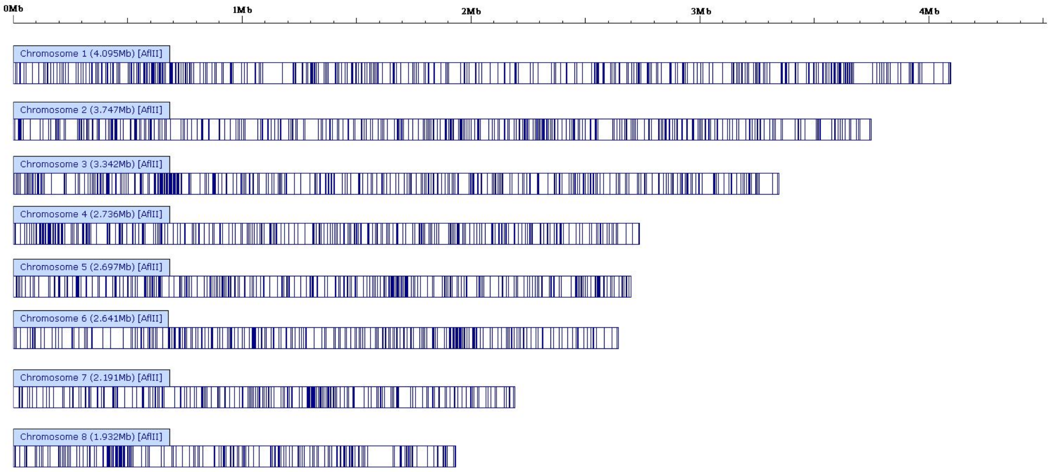

Supplementary Figure S1. An optical map of *M. purpureus* YY-1 consisting of eight chromosomes. Each vertical line represents an *AflII* restriction site. Chromosomes are ranked by size.

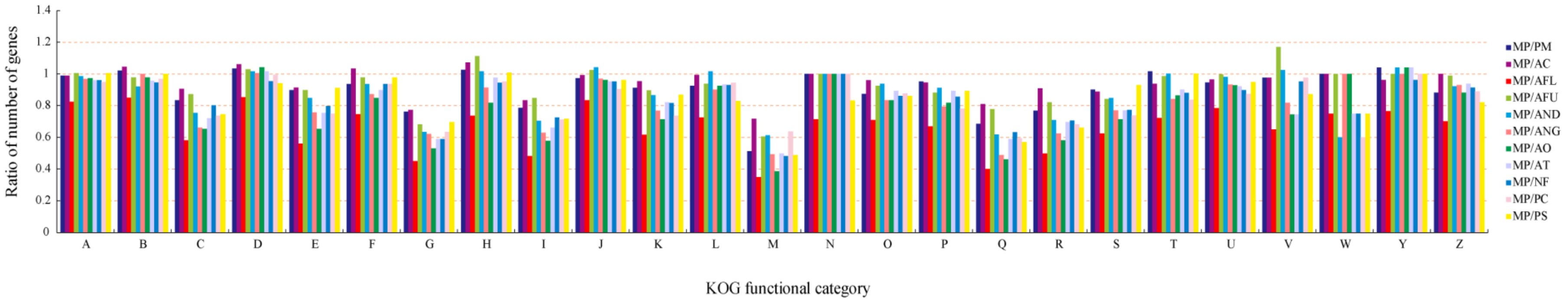

Figure S2. A comparison of relative gene numbers for each KOG. The ratios of the number of genes in *M. purpureus* against those in *P. marneffei* (MP/PM), *A. clavatus* (MP/AC), *A. flavus* (MP/AFL), *A. fumigatus* (MP/AFU), *A. nidulans* (MP/AND), *A. niger* (MP/ANG), *A. oryzae* (MP/AO), *A. terreus* (MP/AT), *N. fischeri* (MP/NF), *P. chrysogenum* (MP/PC) and *P. stipitatus* (MP/PS) for each KOG category were calculated. KOG functional category: A, RNA processing and modification; B, chromatin structure and dynamics; C, energy production and conversion; D, cell cycle control, cell division, chromosome partitioning; E, RNA processing and modification; F, nucleotide transport and metabolism; G, carbohydrate transport and metabolism; H, coenzyme transport and metabolism; I, lipid transport and metabolism; J, translation, ribosomal structure and biogenesis; K, transcription; L, replication, recombination and repair; M, cell wall/membrane/envelope biogenesis; N, cell motility; O, posttranslational modification, protein turnover, chaperones; P, inorganic ion transport and metabolism; Q, secondary

metabolites biosynthesis, transport and catabolism; R, general function prediction only; S, function unknown; T, signal transduction mechanisms; U, intracellular trafficking, secretion, and vesicular transport; V, defense mechanisms; W, extracellular structures; Y, nuclear structure; Z, cytoskeleton.

A

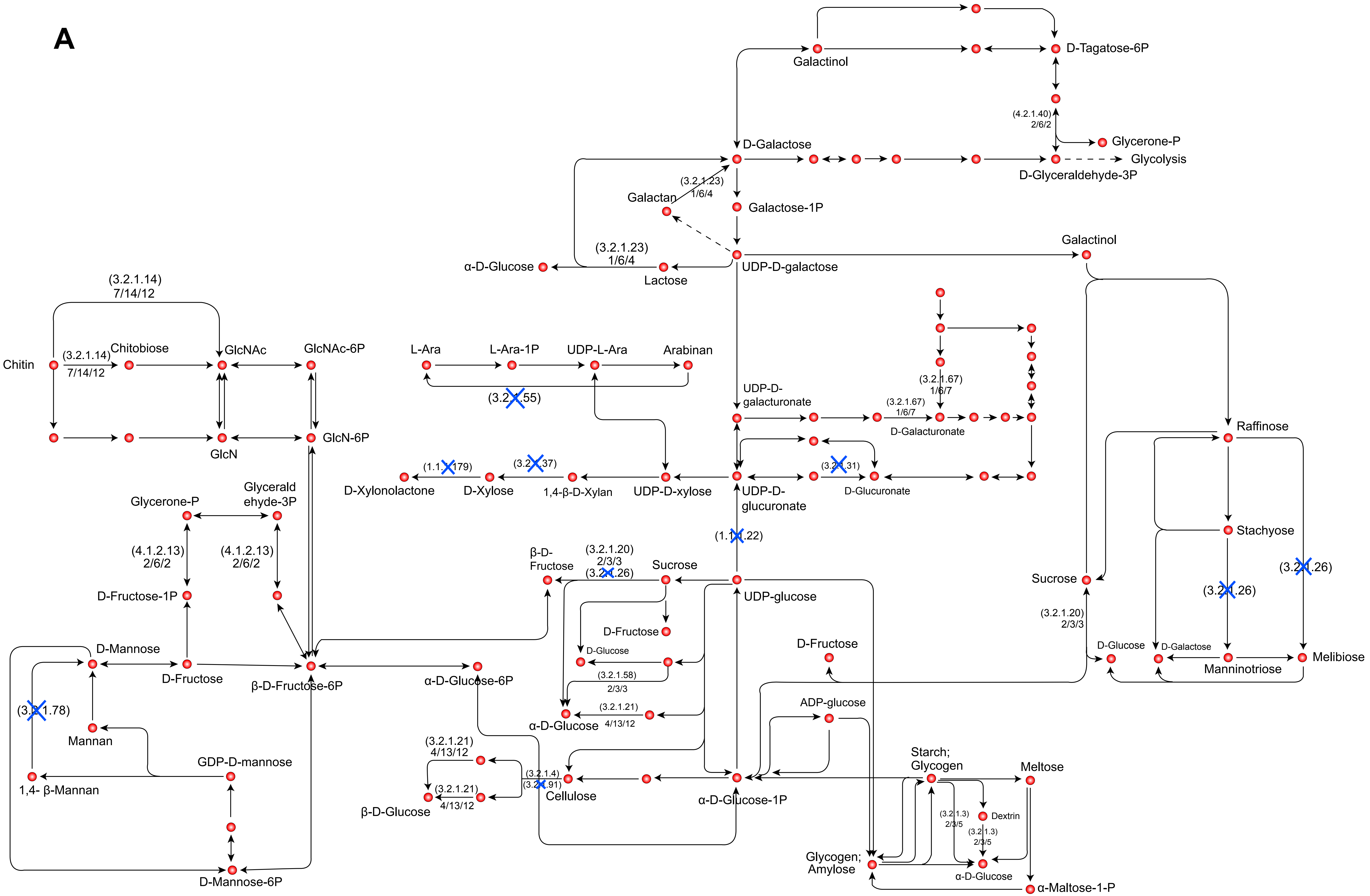

D-Tagatose-6P
Galactinol
(4.2.1.40)
2/6/2
Glycerone-P
D-Galactose
Glycolysis
D-Glyceraldehyde-3P
(3.2.1.23)
1/6/4
Galactan
Galactose-1P
Galactinol
(3.2.1.23)
1/6/4
α-D-Glucose
Lactose
UDP-D-galactose
(3.2.1.14)
7/14/12
Chitin
(3.2.1.14)
7/14/12
Chitobiose
GlcNAc
GlcNAc-6P
L-Ara
L-Ara-1P
UDP-L-Ara
Arabinan
UDP-D-
galacturonate
(3.2.1.67)
1/6/7
(3.2.1.67)
1/6/7
D-Galacturonate
Raffinose
GlcN
GlcN-6P
D-Xylonolactone
D-Xylose
1,4-β-D-Xylan
UDP-D-xylose
UDP-D-
glucuronate
D-Glucuronate
Glycerone-P
Glycerald
ehyde-3P
Stachyose
(4.1.2.13)
2/6/2
(4.1.2.13)
2/6/2
β-D-
Fructose
(3.2.1.20)
2/3/3
Sucrose
Sucrose
D-Fructose-1P
UDP-glucose
(3.2.1.20)
2/3/3
D-Fructose
D-Glucose
D-Fructose
D-Glucose
D-Galactose
Melibiose
Manninotriose
D-Mannose
D-Fructose
β-D-Fructose-6P
α-D-Glucose-6P
(3.2.1.58)
2/3/3
(3.2.1.21)
4/13/12
α-D-Glucose
ADP-glucose
Mannan
GDP-D-mannose
(3.2.1.21)
4/13/12
(3.2.1.4)
Starch;
Glycogen
Meltose
1,4- β-Mannan
(3.2.1.21)
4/13/12
Cellulose
α-D-Glucose-1P
β-D-Glucose
Dextrin
(3.2.1.3)
2/3/5
(3.2.1.3)
2/3/5
Glycogen;
Amylose
α-D-Glucose
D-Mannose-6P
α-Maltose-1-P

B

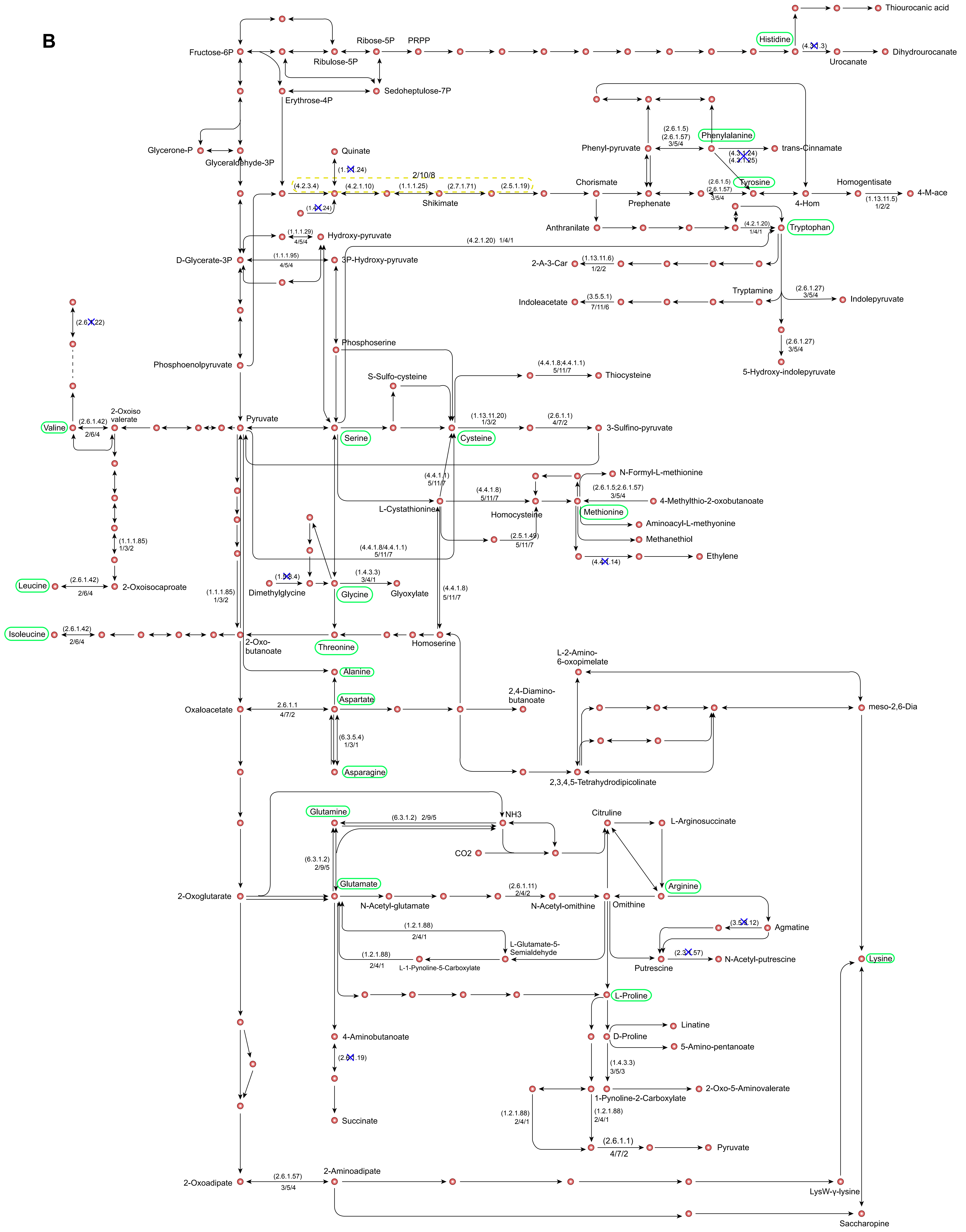

Supplementary Figure S3. A metabolic pathway view of the gene redundancy of pathway members. The gene redundancy of each member of metabolic pathways (A, carbohydrate; B, amino acid) in the *M. purpureus* YY-1 genome compared to *A. oryzae* and *A. fumigatus* is superimposed on the metabolic pathway map. Crosses indicate the missing genes in YY-1. The three values delimited by slashes designate the number of corresponding genes found in the *M. purpureus*, *A. oryzae* and *A. fumigatus* genomes, respectively. The metabolic pathway view was constructed based on the pathways included in KEGG (http://www.genome.ad.jp/kegg/)[54].

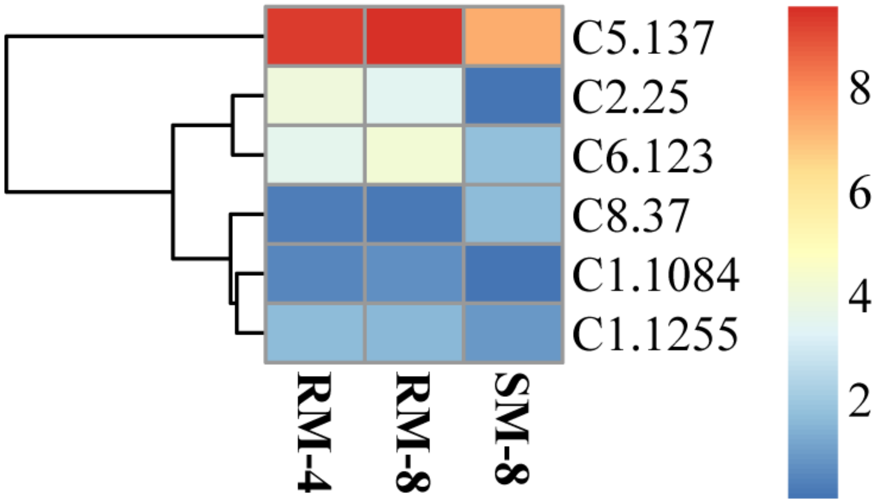

Supplementary Figure S4. Gene expression of six PKS genes in *M. purpureus* YY-1. The tree is a dendrogram of cluster analysis by the hclust method in R, which is a hierarchical partitioning of data that visually resembles a heat map, reflecting gene expression values in different conditions. RM-4 represents *M. purpureus* YY-1 grown in rice medium (RM) on the fourth day, RM-8 represents *M. purpureus* YY-1 grown in rice medium (RM) on the eighth day, and SM-8 represents *M. purpureus* YY-1 grown in sucrose–yeast extract medium (SM) on the eighth day.

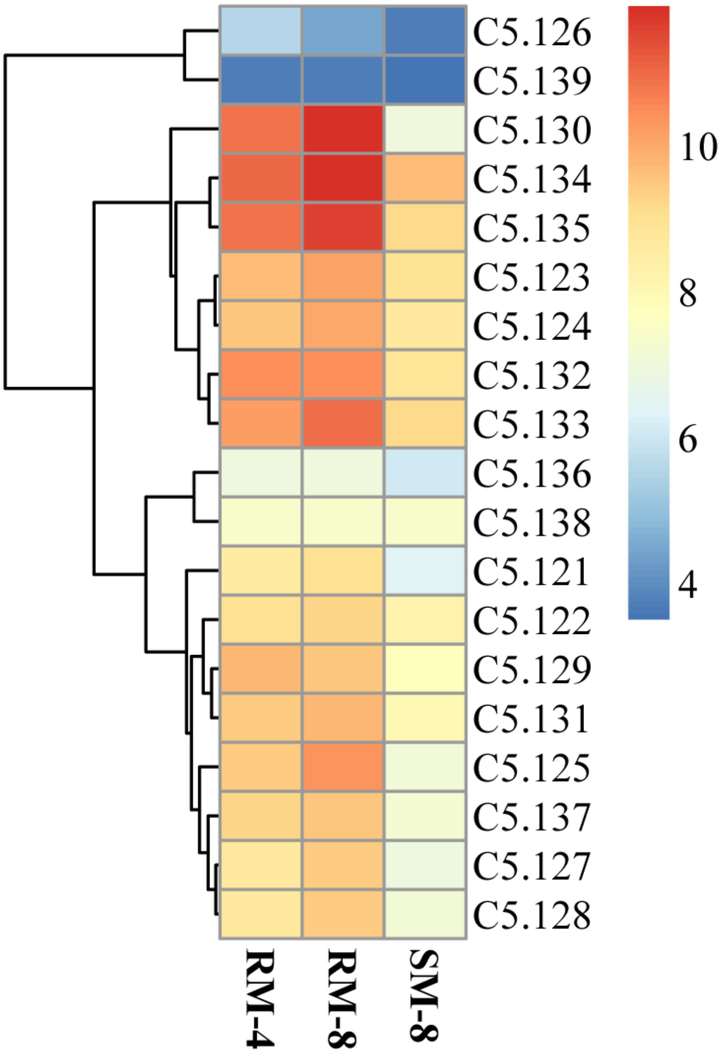

Supplementary Figure S5. Gene expression of the pigment biosynthetic gene cluster in *M. purpureus* YY-1. The tree is a dendrogram of cluster analysis by the hclust method in R, which is a hierarchical partitioning of data that visually resembles a heat map, reflecting gene expression values in different conditions. RM-4 represents *M. purpureus* YY-1 grown in rice medium (RM) on the fourth day, RM-8 represents *M. purpureus* YY-1 grown in rice medium (RM) on the eighth day, and SM-8 represents *M. purpureus* YY-1 grown in sucrose–yeast extract medium (SM) on the eighth day.

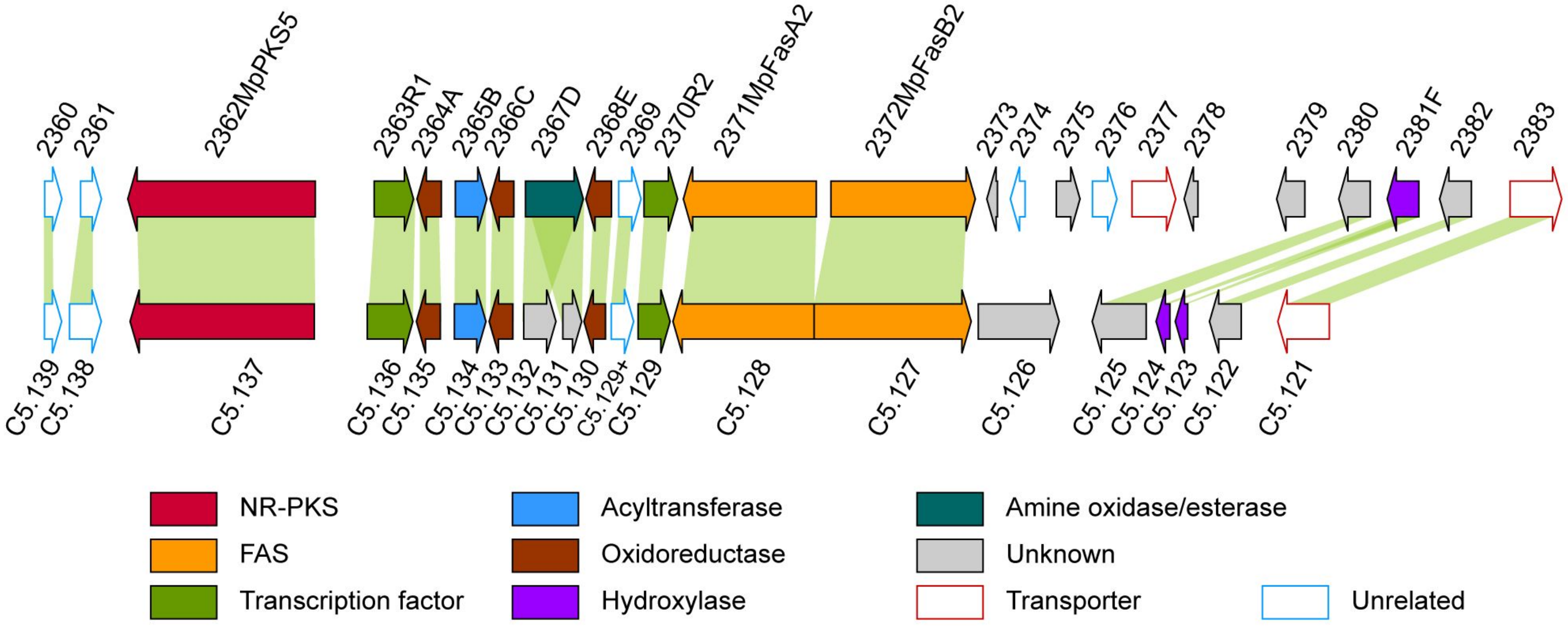

Supplementary Figure S6. Synteny analysis of the pigment biosynthetic gene clusters in *M. pilosus* and *M. purpureus.* The upper panel shows the gene cluster in the *M. pilosus* genome. The bottom panel shows the gene cluster in the *M. purpureus* genome.

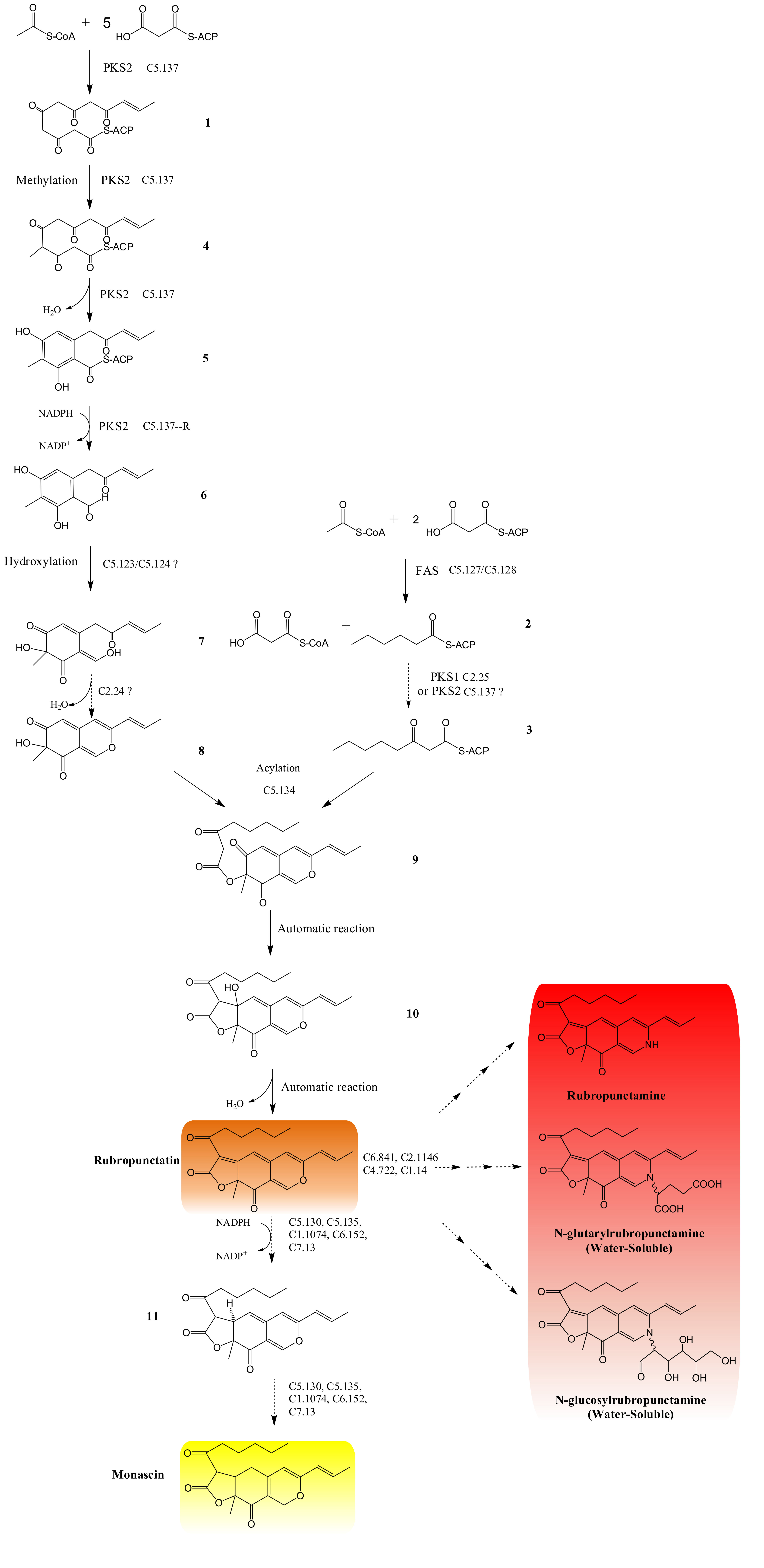

Supplementary Figure S7 The proposed biosynthetic pathway for *Monascus* pigments, including orange pigment (rubropunctatin), yellow pigment (monascin), red pigment (rubropunctamine) and water-soluble pigments. The solid arrows indicate the experimentally identified steps, and the dashed arrows indicate the speculative procedures. In the assembly of the skeleton structure of the pigments, a 12-carbon acyl chain 1 and β-keto group 2 are produced from acetyl-coenzyme A and malonyl-coenzyme A by the PKS2 gene C5.137[57,58] and FAS gene pair C5.127/C5.128[12,13], respectively. We suggest that PKS2 acts as a dual-functioning PKS or that another PKS gene, PKS1 (C2.25), is responsible for the synthesis of compound 3[18]. The formation of the classical polyketide chromophore structure 8 involves methylation[55], cyclization[57], reduction[58], hydroxylation[59] and dehydration, followed by compound 1 formation, successively. In parallel, the condensation product 3 has been proposed to be transferred to polyketide chromophore 8 by acylation from the acyl carrier protein (ACP)-domain to yield 9[13, 59,60]. Then, the intermediate 9 is converted to orange pigment (rubropunctatin) by automatic dehydration. Orange pigments are used as the precursor for yellow and red pigments. The mechanisms by which orange pigments is converted to yellow pigment (monascin) are reduction steps in the existence of NADPH. Red pigment (rubropunctamine) and the water-soluble pigments may undergo further reduction and dehydration followed by amination[61,62].

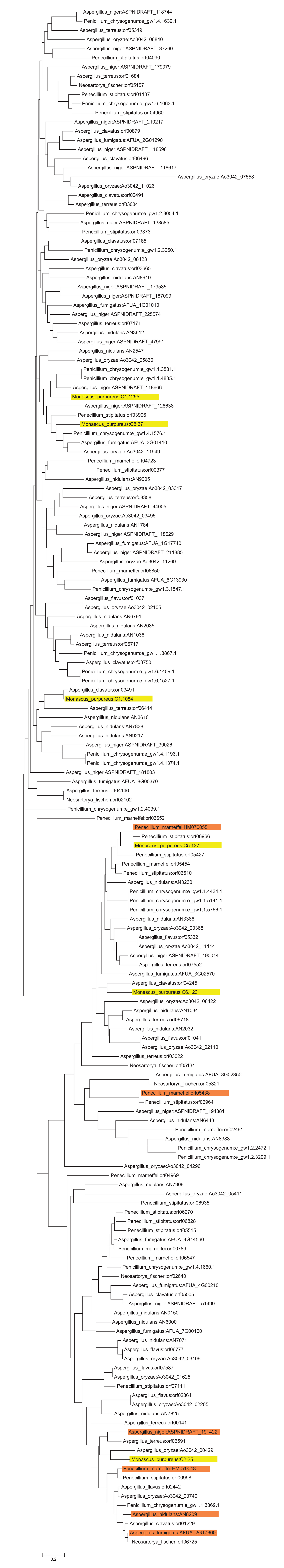

Supplementary Figure S8. Phylogenetic analysis of PKS genes in 12 Eurotiales. PKS genes of *M. purpureus* YY-1 are yellow colored, and the reported pigment-related PKS genes of relatives are orange colored.

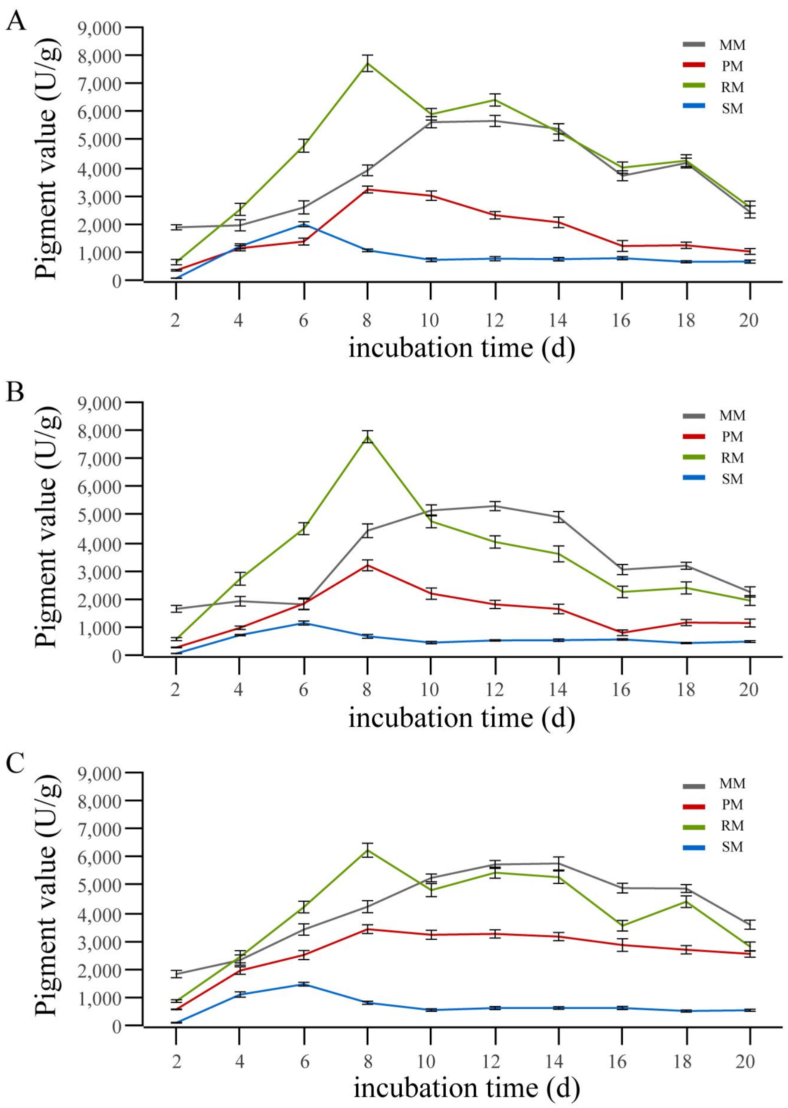

Supplementary Figure S9. Pigment (red, orange and yellow pigments) production by *M. purpureus* YY-1 in four different mediums (malt medium (MM), potato/dextrose broth medium (PM), rice medium (RM) and sucrose–yeast extract medium (SM)) were monitored from the 2nd to the 20th day. All experiments were performed in triplicate. Error bars indicate the standard deviation.

A. Red pigment production by *M. purpureus* YY-1.

B. Orange pigment production by *M. purpureus* YY-1.

C. Yellow pigment production by *M. purpureus* YY-1.

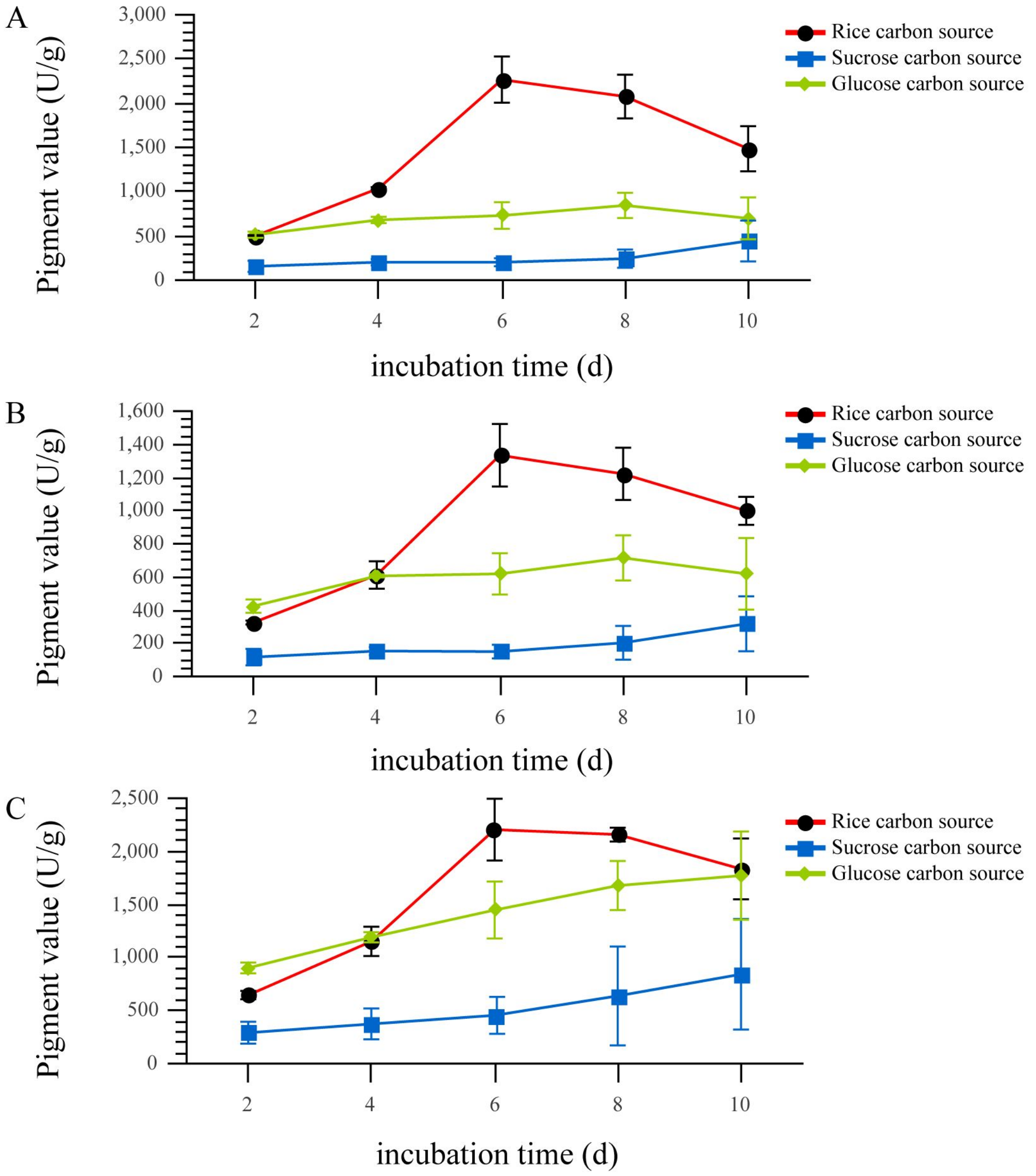

Supplementary Figure S10. Pigment (red, orange and yellow pigments) production in *M. purpureus* YY-1 grown on media with different carbon sources (rice powder, glucose, and sucrose) from the 2nd to 10th days. All experiments were performed in triplicate. Error bars indicate the standard deviation.

A. Red pigment production by *M. purpureus* YY-1.

B. Orange pigment production by *M. purpureus* YY-1.

C. Yellow pigment production by *M. purpureus* YY-1.

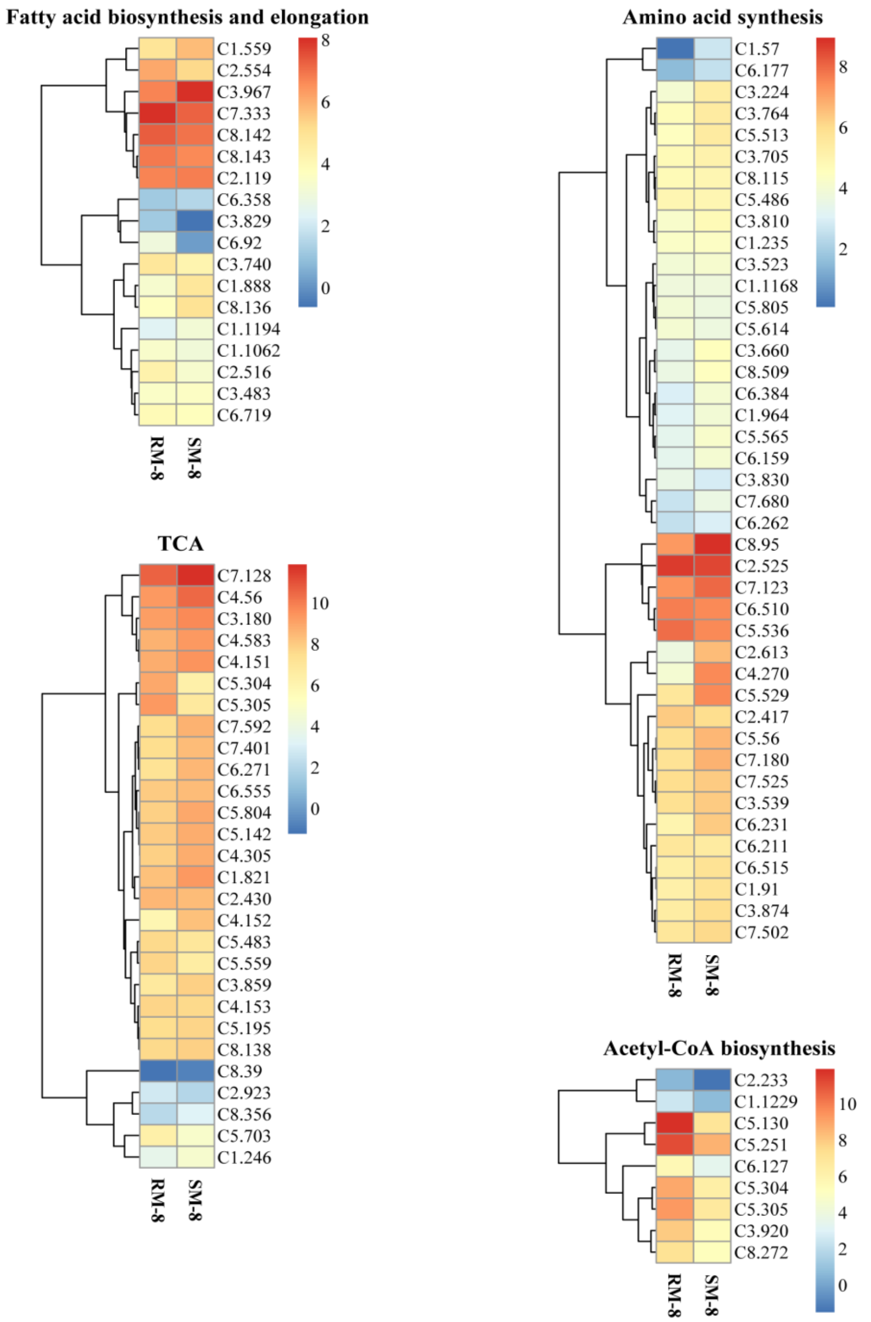

Supplementary Figure S11. Gene expression in pigment biosynthesis-related metabolic pathways in *M. purpureus* YY-1 grown on different carbon sources. The tree is a dendrogram of cluster analysis by the hclust method in R, which is a hierarchical partitioning of data that visually resembles a heat map, reflecting

gene expression values in different conditions. RM-8 represents *M. purpureus* YY-1 grown in rice medium (RM) on the eighth day, and SM-8 represents *M. purpureus* YY-1 grown in sucrose–yeast extract medium (SM) on the eighth day.

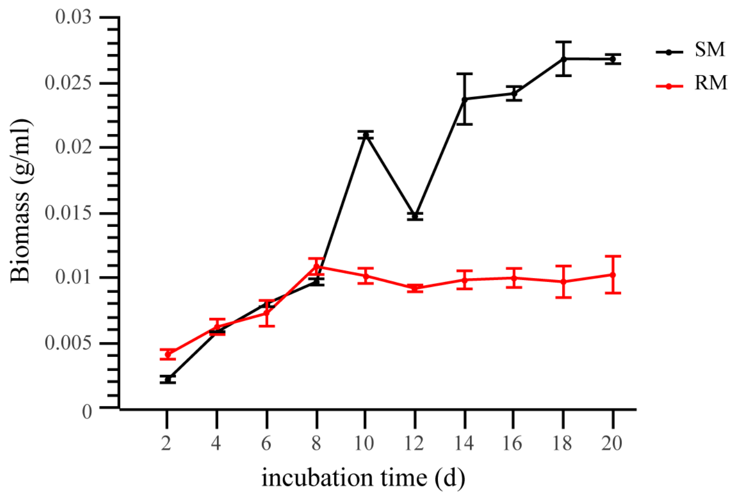

Supplementary Figure S12. Biomass (dry cell weight) of M. purpureus YY-1 grown in rice medium (RM) and sucrose–yeast extract medium (SM) were monitored from the 2nd to 20th day. All experiments were performed in triplicate. Error bars indicate the standard deviation.

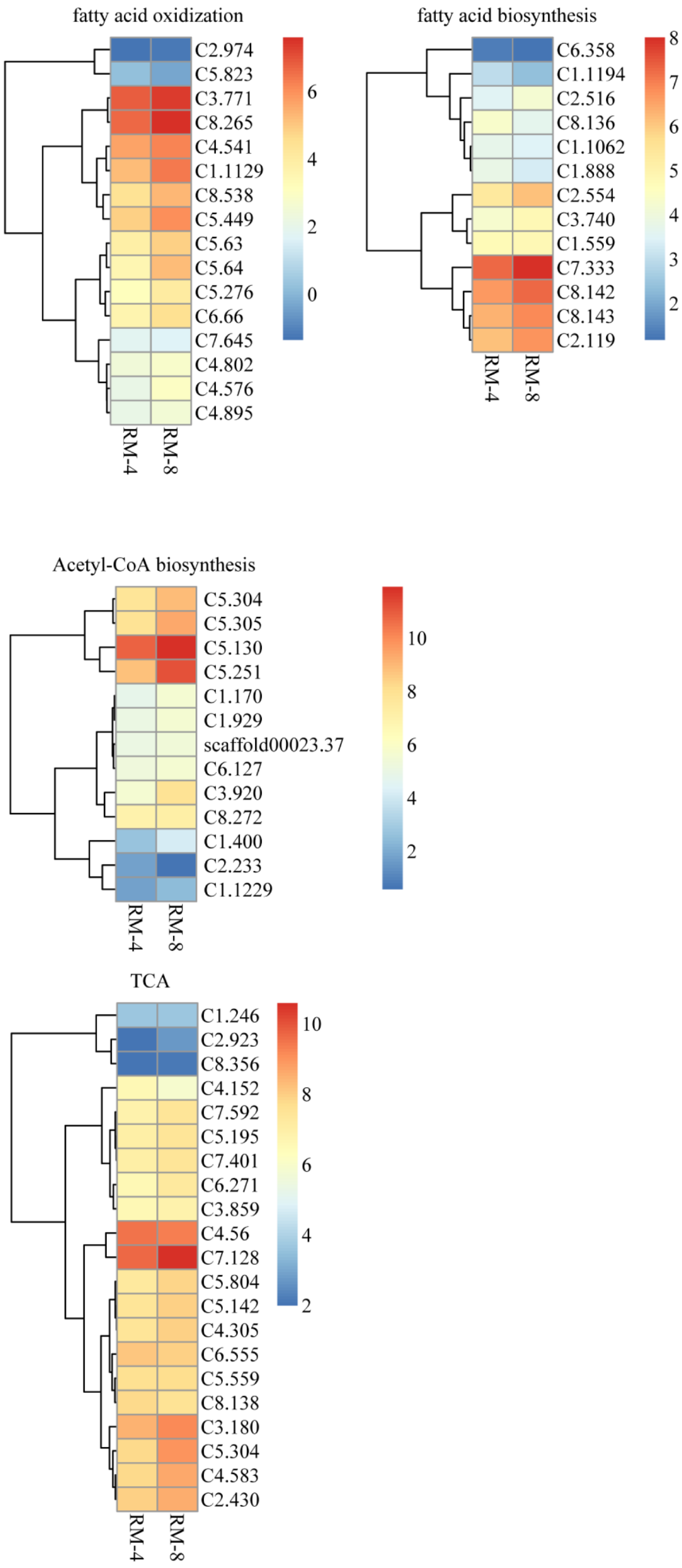

Supplementary Figure S13. Gene expression in pigment biosynthesis-related metabolic pathways in *M. purpureus* YY-1 at different grown stages in rice

medium. The tree is a dendrogram of cluster analysis by the hclust method in R, which is a hierarchical partitioning of data that visually resembles a heat map, reflecting gene expression values in different conditions. RM-4 represents *M. purpureus* YY-1 grown in rice medium (RM) on the fourth day, and RM-8 represents *M. purpureus* YY-1 grown in rice medium (RM) on the eighth day.

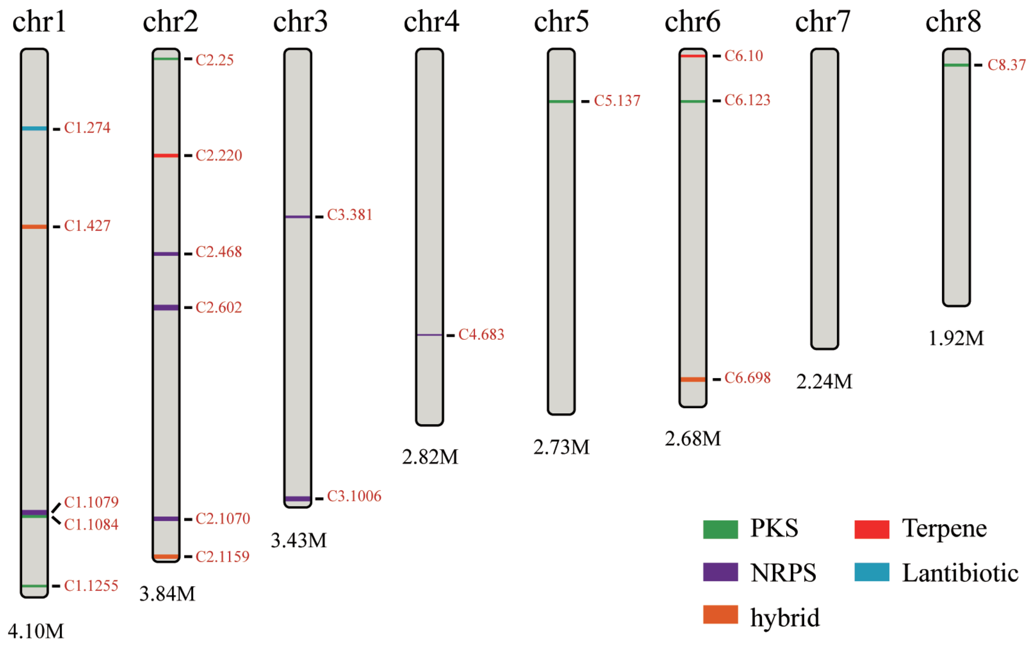

Supplementary Figure S14. The location of secondary metabolic genes in M. purpureus YY-1. Secondary metabolic genes predicted using the antiSMASH program are marked with different colors.

A

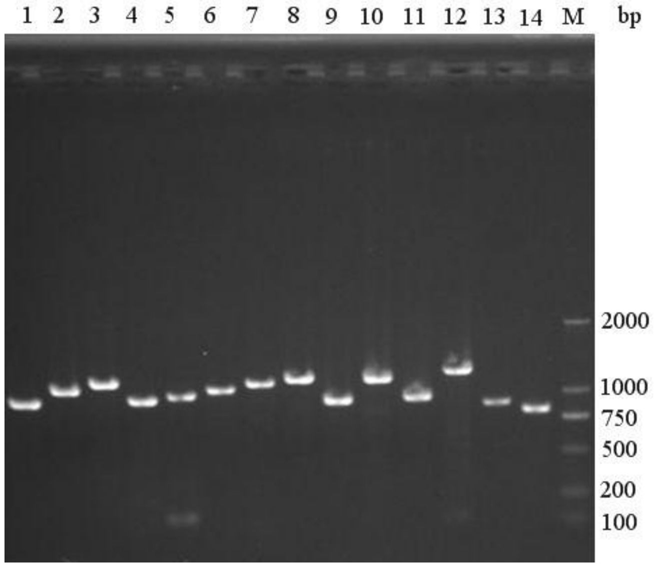
1 2 3 4 5 6 7 8 9 10 11 12 13 14 M
bp
2000
1000
750
500
200
100

B

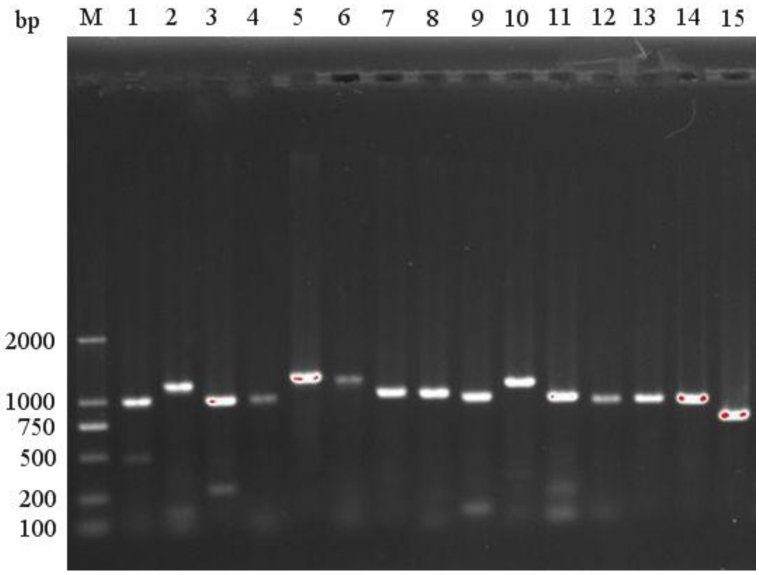
bp
M 1 2 3 4 5 6 7 8 9 10 11 12 13 14 15
2000
1000
750
500
200
100

C

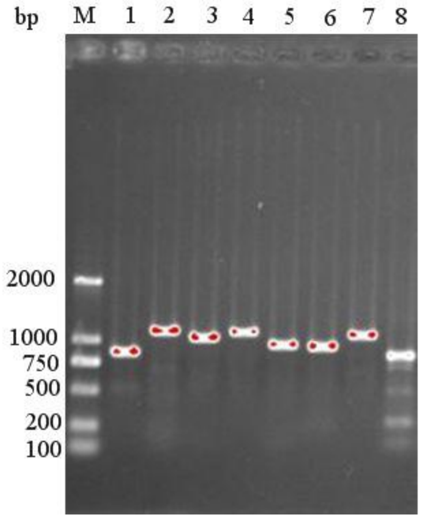

Supplementary Figure S15. Agrose gel electrophoresis of PCR products which confirms the presence of PKS-NRPS hybrids (C1.427, C2.1159 and C6.698) in *M. purpureus* YY-1. Specific DNA fragments with expected size (Supplementary Data S8) could be amplified from (A) C1.427, (B) C2.1159 and (C) C6.698, respectively.

Supplementary References

54 Kanehisa, M., Goto, S., Furumichi, M., Tanabe, M. & Hirakawa, M. KEGG for representation and analysis of molecular networks involving diseases and drugs. *Nucleic Acids Res.* **38**, D355-D360 (2010).

55 Cox, R. J. Polyketides, proteins and genes in fungi: programmed nano-machines begin to reveal their secrets. *Organic & Biomolecular Chemistry* **5**, 2010-2026 (2007).

56 Crawford, J. M. *et al.* Deconstruction of iterative multidomain polyketide synthase function. *Science* **320**, 243-246 (2008).

57 Crawford, J. M. *et al.* Structural basis for biosynthetic programming of fungal aromatic polyketide cyclization. *Nature* **461**, 1139-1143 (2009).

58 Bailey, A. M. *et al.* Characterisation of 3-methylorcinaldehyde synthase (MOS) in *Acremonium strictum*: first observation of a reductive release mechanism during polyketide biosynthesis. *Chem. Commun.*, 4053-4055 (2007).

59 Zabala, Angelica O., Xu, W., Chooi, Y.-H. & Tang, Y. Characterization of a silent azaphilone gene cluster from *Aspergillus niger* ATCC 1015 reveals a hydroxylation-mediated pyran-ring formation. *Chem. Biol.* **19**, 1049-1059 (2012).

60 Xie, X., Meehan, M. J., Xu, W., Dorrestein, P. C. & Tang, Y. Acyltransferase mediated polyketide release from a fungal megasynthase. *J. Am. Chem. Soc.* **131**, 8388-8389 (2009).

61 Lin, T. F., Yakushijin, K., Büchi, G. H. & Demain, A. L. Formation of

water-soluble *Monascus* red pigments by biological and semi-synthetic processes. *J. Ind. Microbiol.* **9**, 173-179 (1992).

62 Hajjaj, H. *et al.* Production and identification of N-glucosylrubropunctamine and N-glucosylmonascorubramine from *Monascus ruber* and occurrence of electron donor-acceptor complexes in these red pigments. *Appl. Environ. Microbiol.* **63**, 2671-2678 (1997).
